# Neonatal brain connectivity outliers identify over forty percent of IQ outliers at 4 years of age

**DOI:** 10.1002/brb3.1846

**Published:** 2020-09-17

**Authors:** Wei Gao, Yuanyuan Chen, Emil Cornea, Barbara D. Goldman, John H. Gilmore

**Affiliations:** ^1^ Department of Biomedical Sciences and Imaging Biomedical Imaging Research Institute (BIRI) Cedars‐Sinai Medical Center Los Angeles CA USA; ^2^ Department of Medicine University of California at Los Angeles Los Angeles CA USA; ^3^ Department of Psychiatry University of North Carolina Chapel Hill Chapel Hill NC USA; ^4^ Department of Psychology and Neuroscience FPG Child Development Institute University of North Carolina Chapel Hill Chapel Hill NC USA

## Abstract

**Background:**

Defining reliable brain markers for the prediction of abnormal behavioral outcomes remains an urgent but extremely challenging task in neuroscience research. This is particularly important for infant studies given the most dramatic brain and behavioral growth during infancy.

**Methods:**

In this study, we proposed a novel prediction scheme through abstracting individual newborn's whole‐brain functional connectivity pattern to three outlier measures (Triple O) and tested the hypothesis that neonates identified as “brain outliers” based on Triple O were more likely to develop as IQ outliers at 4 years of age. Without need for training with behavioral data, Triple O represents a novel proof‐of‐concept approach to predict later IQ outcomes based on neonatal brain data.

**Results:**

Triple O correctly identified 42.1% true IQ outliers among a mixed cohort of 175 newborns with different term, twin, and maternal disorder statuses. Triple O also reached a high level of specificity (96.2%) and overall accuracy (90.3%). Further incorporating a demographic information indicator, the enhanced Triple O+ could further differentiate between high and low 4YR IQ outliers. Validation tests against seven independent reference samples revealed highly consistent results and a minimum sample size of ~50 for robust performance.

**Conclusions:**

Considering that postnatal brain growth and various environmental factors likely also contribute to 4YR IQ, the fact that Triple O, based purely on neonatal functional connectivity data, could identify >40% of 4YR IQ outliers is striking. Together with the very high level of specificity, each outlier predicted by Triple O represents a meaningful risk but future efforts are needed to explore ways to identify the rest of outliers. Overall, with no need for training, a high level of robustness, and a minimal requirement on sample size, the proposed Triple O approach demonstrates great potential to predict later outlying IQ performances using neonatal functional connectivity data.

## Graphical abstract

One of the most desired goals of developmental research is to be able to predict childhood behavioral outcomes based on brain biomarkers as early as possible to facilitate in‐time intervention. However, this problem is notoriously challenging, partly due to the complex nature of brain function and reportedly weak brain–behavioral relationships. In this study, we derived a simple, training‐free prediction scheme to abstract the neonatal whole‐brain functional connectivity pattern to three outlier measures (Triple O) and showed that it could identify over forty percent of 4‐year IQ performance outliers with a high level of robustness against different reference samples. Results in this study represent a first step in this novel direction of brain outlier‐based prediction of later behavioral outcomes.

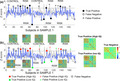

## 1 INTRODUCTION

Over the past two decades, the successful application of resting‐state functional magnetic resonance imaging (rsfMRI) technique (Biswal, Yetkin, Haughton, & Hyde, 1995) in the infant population has dramatically improved our understanding of the fast‐paced, nonlinear, and patterned development of the brain's functional networks during the first years of life (Doria et al., 2011; Fransson, Aden, Blennow, & Lagercrantz, 2011; Gao, Lin, Grewen, & Gilmore, 2016; Gao et al., 2009; Smyser et al., 2010; Thomason et al., 2013). Plastic and modifiable by a range of beneficial and risk factors (Gao et al., 2016), functional brain development during this period harbors both great opportunity and vulnerability, consistent with the developmental origins of health and disease (DOHaD) hypothesis (Monk, Lugo‐Candelas, & Trumpff, 2019; Silveira, Portella, Goldani, & Barbieri, 2007). Therefore, one of the most important goals for developmental imaging research is to explore whether and how early brain‐based biomarkers could identify risks for later adverse developmental outcomes. To this aim, promising associations between early brain functional connectivity and later behavioral outcomes have been reported (Alcauter, Lin, Smith, Short, et al., 2014; Ball et al., 2015; He et al., 2018; Salzwedel et al., 2019; Strahle et al., 2019), supporting a positive answer to the “if” question but “how” exactly accurate prediction can be achieved remains elusive.

Due to the interconnected, coordinated, and network nature of brain function (Achard & Bullmore, 2007; Bressler, 1995), one potential barrier underlying the brain–behavioral prediction challenge relates to a “many‐to‐one” problem as there are likely a wide range of brain processes involved in any single behavioral output (Atzil, Gao, Fradkin, & Barrett, 2018; Atzil, Hendler, & Feldman, 2014; Lindquist, Wager, Kober, Bliss‐Moreau, & Barrett, 2012; Song et al., 2008). Supporting this notion, previous reports on brain–behavioral relationships in infants often report weak to moderate correlations between a number of functional connections and a single behavioral output (Alcauter, Lin, Smith, Short, et al., 2014; He et al., 2018; Salzwedel et al., 2019), suggesting that individual functional connections/processes likely only contribute a small percentage of variance to a given behavioral phenotype. Adding to the challenge, the collection of functional connections/processes may show both positive and negative associations with the same behavioral output and may act in both additive and interactive ways, making efforts to combine them for prediction even more challenging. Intuitively, these issues may seem to suggest advanced machine learning‐based algorithms as a suitable solution but the need for “big data” for training of such algorithms make them impractical at this point, given the sparsity of available data due to inherent challenges in research‐based infant MRI imaging.

An alternative solution lies in hypothesis‐driven prediction through informed feature selection and dimension reduction. The key for the success of such approaches lies in the way of abstraction so that the selected features could capture the most salient information that are predictive of the later behavioral output in question. In this paper, we sought to derive a novel abstraction‐prediction framework based on neonatal functional connectivity pattern and test its predictive values for 4‐year IQ (4YR IQ) performance. As a composite measure of general cognitive capability, IQ covers multiple functional domains including fluid reasoning, verbal and nonverbal knowledge, quantitative skills, visual–spatial processing, and working memory (Roid, 2003). Each of these functional domains is supported by a distributed set of brain regions and/or networks; thus, the brain basis of IQ is likely represented by a complex whole‐brain system composed of widely distributed but coordinating functional networks (Goriounova & Mansvelder, 2019). Given this integrated and global nature of the potential brain basis of IQ, we propose a “Triple Outliers (Triple O)” approach to reduce the neonatal whole‐brain functional connectivity pattern into three global outlier measures and applied them to 175 study participants to categorically predict their 4‐year IQ performances as being or not being an “outlying” performer. Given the DOHaD (Monk et al., 2019; Silveira et al., 2007) and the developmental cascading hypothesis (Masten & Cicchetti, 2010), we hypothesize that newborns characterized as “global brain outliers” would more likely manifest later outlying IQ performances. This hypothesis is empirically supported by previous studies reporting a number of functional connections showing linear correlations with composite cognitive outcomes later in life (Alcauter, Lin, Smith, Goldman, et al., 2014; Alcauter, Lin, Smith, Short, et al., 2014; Salzwedel et al., 2019). These studies suggest that subjects more frequently lying on the two ends of the functional connectivity continuum should also be more likely to appear at the two ends of the behavioral spectrum. We propose sign‐insensitive outlier measures in Triple O since both positive and negative associations at the individual connection level have been observed (Alcauter, Lin, Smith, Goldman, et al., 2014; Alcauter, Lin, Smith, Short, et al., 2014; Salzwedel et al., 2019). Similarly, the sign‐insensitive nature of Triple O may also mitigate the recently reported heterogeneity in brain–behavior relationships within the neonate population (Chen et al., 2020) through equally accounting the absolute deviations from the population mean regardless of the different directions of brain–behavior associations in different subgroups. Note, however, there are likely other postnatal brain (both structural and functional) and environmental factors (e.g., parenting, family environment/enrichment activity, adverse life events, and nutrition) that also contribute to 4YR IQ outcomes so we do not expect the proposed Triple O to capture all 4YR IQ outliers. Overall, as a novel hypothesis‐driven prediction approach with no need for training with IQ outcome data, we do anticipate the proposed Triple O approach and its future extensions to have high translational potential to aid clinical identification of newborns at risk for adverse developmental outcomes later in life due to abnormal functional brain growth before the neonatal stage.

## 2 MATERIALS AND METHODS

### 2.1 Participants and Image Acquisition

Three hundred and ninety three infant participants were retrospectively identified from the UNC‐Chapel Hill Early Brain Development Study, characterizing early childhood brain and behavior development (Gao et al., 2016; Gilmore, Knickmeyer, & Gao, 2018). Based on whether there was an available 4‐year‐old IQ (4YR IQ) score, these infants were separated into two groups: SAMPLE 1 (*N* = 175) with both available neonate MRI scans and 4‐year‐old IQ scores, and SAMPLE 2 (*N* = 218) with only available neonate MRI scans but no 4YR IQ. Both SAMPLE 1 and SAMPLE 2 were composed of mixed samples with respect to sex, term status (i.e., either full‐term or preterm), twin status (i.e., either singleton or twins), and maternal mental disorder status (i.e., with or without maternal disorder diagnosis, including schizophrenia, bipolar disorder, and other nonspecified psychiatric disorders). Besides these three categorical variables, we also have continuous measures of gestational age (GA) at birth, GA at MRI scan/4YR IQ assessment, birthweight, birth length, maternal/paternal age, maternal/paternal education in years, and total annual family income. The detailed demographic information for both SAMPLE 1 and SAMPLE 2 is listed in Table 1. These heterogeneous samples were selected to enrich developmental outcomes as well as to test the practical applicability of our prediction approach.

**Table 1:** Demographic information of SAMPLE 1 and SAMPLE 2 and their comparisons

	SAMPLE 1 (*N* = 175)	SAMPLE 2 (*N* = 218)	*p*‐values comparing SAMPLE 1/2
Sex (male/female)	87/88	108/110	.973
Twin status (single/twin birth)	81/94	81/137	.068
Term status (full‐term/preterm)	112/63	130/88	.378
Maternal disorder diagnosis (disorder/no disorder)	21/154	30/188	.607
Scanner (1/2)	149/26	181/37	.451
Gestational age at birth (days)	261 ± 18.27	259.15 ± 20.66	.353
Gestational age at scan (days)	293.93 ± 13.91	294.47 ± 14.82	.714
Maternal education (years)	15.3 ± 3.33	14.95±±3.84	.340
Paternal education (years)	15.10 ± 3.28	15.07 ± 3.84	.944
Maternal age at birth (years)	30.13 ± 6.03	29.64 ± 5.63	.403
Paternal age at birth (years)	32.69 ± 6.73	32.33 ± 6.74	.606
Birth weight (g)	2,805.61 ± 685.42	2,720.80 ± 704.43	.231
Birth length (mm)	48.23 ± 3.89	47.97 ± 3.76	.522
Total household income ($)	74,845.72 ± 56,303.35	74,489.76 ± 60,177.71	.954

IQ scores at 4 years of age were measured using the Stanford–Binet Intelligence Scales, 5th edition (Roid, 2003). The Stanford–Binet is a series of tasks administered individually in a structured setting. These scales were designed to assess intelligence across the life span, with focuses on five major domains, including fluid reasoning, knowledge, quantitative, visual–spatial processing, and working memory. In the current study, the Abbreviated IQ (ABIQ) score was used as a measure of general cognitive ability. This IQ score is calculated from performance on two routing subtests: a nonverbal test involving object or sequence/pattern recognition and a verbal test of vocabulary. The Abbreviated IQ score provides a quick estimate of a child's general cognitive ability and as it requires the administration of only two subtests. Therefore, it is easier to obtain than the full‐scale IQ, especially for 4‐year‐old children. The ABIQ score has shown strong test–retest (*r* = .87) reliability. The Stanford–Binet scales also have strong interrater reliability (ranging from 0.74 to 0.97 across all scales). The overall study protocols were approved by both the UNC at Chapel Hill and Cedars‐Sinai Institutional Review Boards.

MRI data were acquired using two scanners: a 3T Siemens Allegra scanner with a circular polarization head coil (330 neonatal scans) and a 3T Siemens Tim Trio with a 32‐channel head coil (63 neonatal scans). Functional images were acquired with a T2* weighted echo planar imaging (EPI) sequence: TR/TE = 2,000 ms/ 32 ms, 33 slices, voxel size = 4 mm^3^, 150 volumes of repetition. Structural images were acquired using a 3D MPRAGE sequence: TR/TE = 1,820 ms/ 4.38 ms, TI = 1,100 ms, voxel size = 1 mm^3^. Infant subjects were fed, swaddled, and fitted with ear protection prior to imaging. All subjects were in a natural sleep state during the imaging session.

### 2.2 Image processing

Functional images were preprocessed using the FMRIB's Software Libraries (FSL) (Smith et al., 2004) and AFNI (Cox, 1996), including discarding the first 10 volumes, slice timing and head motion correction, band‐pass filtering (0.01–0.08 Hz), and nuisance signal regression. The 24 motion‐related parameters (six motion correction parameters, derivative, and their quadratic terms), white matter, CSF, and global signals (also including their derivative and quadratic terms) were included as nuisance signals. All the nuisance signals were band‐pass‐filtered (0.01–0.08 Hz) before regression to match the frequency of the BOLD signal. Data scrubbing was performed as an added motion correction step in addition to the standard rigid‐body motion correction procedures. Specifically, volumes with global signal changes >0.5% and/or framewise displacements (FD) >0.3 mm (Power et al., 2014) were excluded (plus one before and two after). Subjects with <90 volumes (=3 min) were excluded from the study. After functional images preprocessing, all functional images were registered to the age‐specific anatomical template space (Shi et al., 2011) for each age group using the combined transformation field from a two‐step registration, namely an affine transformation from individual functional images to anatomical images and a nonlinear registration from individual anatomical images to the target images. Spatial transformations were performed in FSL. The amount of volumes scrubbed and residual framewise FD (rFD) were compared cross‐sectionally to ensure there were no differences in motion and included as motion covariates in statistical analysis. Finally, the images were spatially smoothed with Gaussian kernel (FWHM = 6 mm) and truncated into 90 volumes to increase consistency across subjects. Finally, UNC‐CEDARS functional parcellation atlas for neonate brains (Shi, Salzwedel, Lin, Gilmore, & Gao, 2017), including 223 regions, was used to create the whole‐brain functional connectivity matrix. One region was lost when downsampling into 4‐mm spatial resolution resulting in a final matrix size of 222 × 222 for each subject. The correlation of fMRI signals between paired regions was Fisher‐*Z*‐transformed for subsequent analysis.

### 2.3 IQ outlier detection

**Figure 1:**
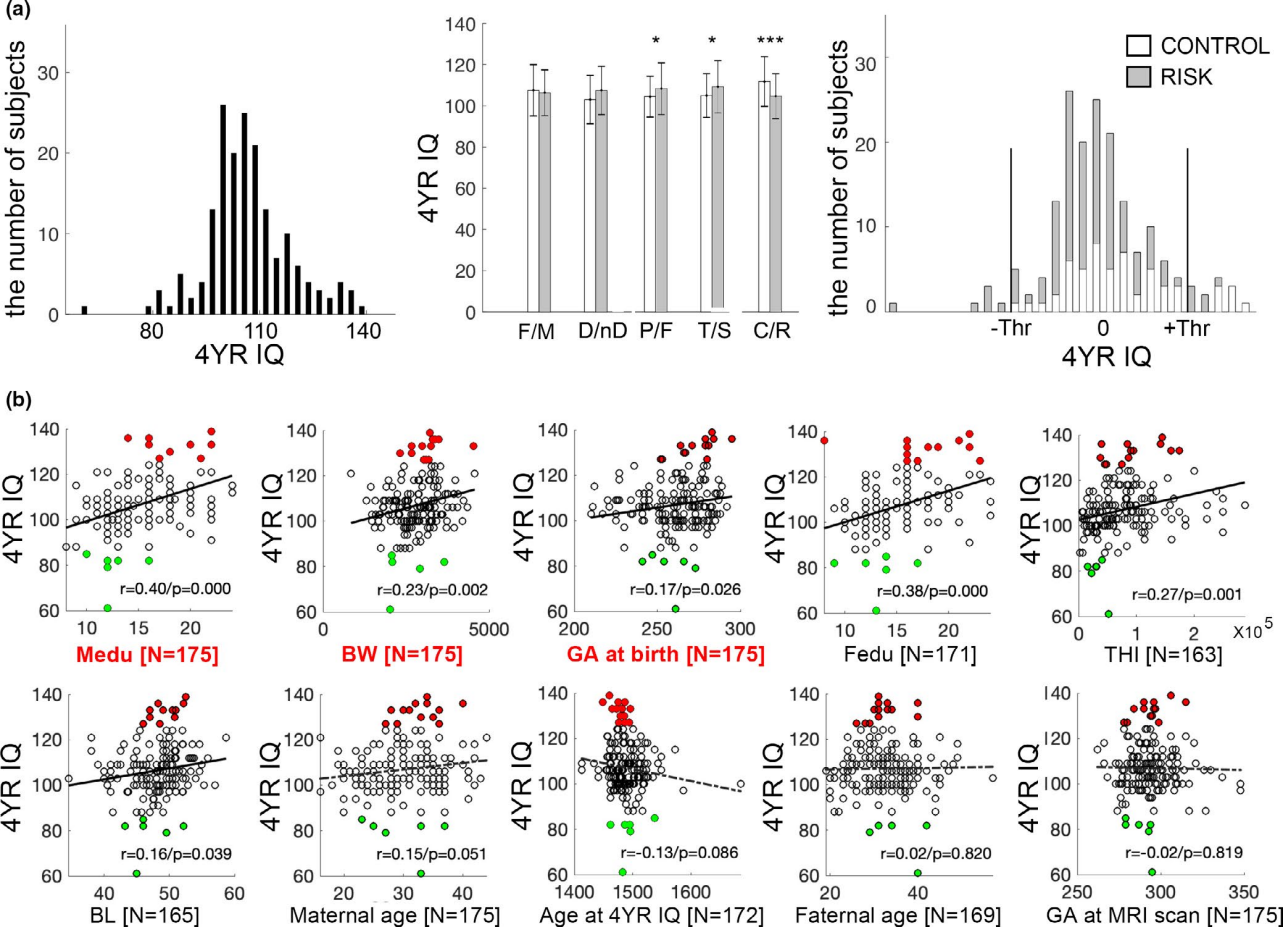
Distribution of 4‐year IQ (4YR IQ) scores across different groups and correlations with demographic variables. (a) Left: 4YR IQ histogram of SAMPLE 1 (*N* = 175); middle: comparisons of 4YR IQ between males and females (M/F), subjects with and without maternal disorder (D/nD), full‐term and preterm (P/F), and twins/singletons (T/S) within SAMPLE 1. The right most bar represents the comparison between the composite CONTROL group (i.e., full‐term, singleton birth, and no maternal disorder diagnosis) and the RISK group (i.e., subjects with one of the three risk factors including twin, preterm birth, and maternal disorder diagnosis. (C/R); right: comparison of 4YR IQ histograms within the CONTROL and RISK subsamples of SAMPLE 1 (vertical lines indicating the *p* < .05 lines used to operationally define 4YR IQ outliers in this study). (b) Scatter plots showing correlations between all continuous demographic variables and 4YR IQ. Solid black regression lines indicate significant correlations, while those dashed lines do not. The high/low outlying 4YR IQ performers as shown in (a) right panel were highlighted in all scatter plots as red/green dots to show their distribution against each and every demographic variable. The three demographic variables on which we have data on all 175 subjects of SAMPLE 1 and show significant correlations (i.e., maternal education (Medu), birthweight (BW), and gestational age at birth (GA at birth)) were highlighted and listed in the first three panels. Gestational age at birth (GA at birth), gestational age at IQ assessment (GA at 4YR IQ), gestational age at MRI scan (GA at MRI scan), birthweight, birth length, maternal/paternal age, maternal (Medu)/paternal education (Pedu) in years, and total annual household income (THI)

The 4‐year‐old IQ scores were normalized into *Z*‐scores (*Z*_IQ). A one‐tailed significance of *p* = .05 (i.e., *Z* > 1.645) was selected as the threshold to define positive (*Z*_IQ > 1.645) and negative behavior outliers (*Z*_IQ < −1.645). There are 13 positive and 6 negative outliers detected in SAMPLE 1 (Figure 1a).

### 2.4 Brain outlier detection

To define brain outliers, we propose to abstract the whole‐brain 222 × 222 functional connectivity matrix to three simple measures quantifying three different, but related, aspects of being “outlying” against a reference population. The first measure directly counts the number of “outlying” connections against the reference group mean. Similar to IQ outlier definition, a one‐tailed significance of *p* < .05 was used as the threshold to define connection‐level outliers. The rationale for the first measure is intuitive and straightforward; if multiple functional connections linearly correlate with a later behavioral outcome, then the number of times a particular subject sits on the extreme of these connections would likely be associated with the chance of the subject appearing on the extreme of the behavioral outcome given the underlying linear relationships. In other words, the number of “outlying” connections may be viewed as an index of the number of “at‐risk” brain features that may contribute to later abnormal IQ outcomes. Therefore, the number of “outlying” connections was chosen as the first measure. Considering that there could be other subthreshold deviations from the group mean, a second measure was defined as the average Euclidean distance of one subject's vectorized connectivity matrix to every subject's vectorized matrix in the reference group, which summarized the overall deviation of one subject's whole‐brain connectivity pattern to the reference group regardless of any threshold. The second measure represents a complementary measure to the first one to further include those subjects that may show connection‐level subthreshold deviations from the group mean but the sum of which is sufficient to put them at the extreme at the whole‐brain level. Since both the first two measures evaluate subject‐level deviations from the group mean, we proposed another within‐subject measure‐the standard deviation across all individual connections as a third within‐subject measure with the expectation that subjects showing extreme level of functional connectivity variability across the whole brain may also be more likely to manifest as IQ outliers.

We chose to use these whole‐brain level outlier measures in the proposed approach since both positive and negative associations at the individual connection level have been observed (Alcauter, Lin, Smith, Short, et al., 2014; He et al., 2018; Salzwedel et al., 2019). Through identifying global “outliers” relative to the group mean, the proposed Triple O approach would summarize extreme deviations from the population mean regardless of whether they are at the low or high end of the functional connectivity strength spectrums. Therefore, Triple O is not sensitive to positive or negative associations; rather, it “harmonizes” and summarizes absolute deviations from the population mean in both types of brain–behavioral associations for better prediction. More importantly, recent evidences (Chen et al., 2020) suggest that even within an otherwise “homogenous” neonate population, different subgroups of neonates may possess qualitatively different brain–behavior associations (i.e., in different directions). A typical way to address such relational heterogeneity in prediction would likely require subgroup‐specific prediction models to better utilize the differential information. However, the proposed global brain outlier‐based Triple O approach would mitigate such heterogeneity since regardless of the different directions/sings of brain–behavior correlations, the outlier measure will equally capture the absolute deviations of a subject with respect to the group mean for effective prediction.

After the calculation of all three measures, they were normalized to *Z*‐scores based on the mean and standard deviations from the reference group and any values >1.645 standard deviations above the mean (i.e., one‐tailed *p* < .05) were defined as an “outlier.” Therefore, each subject would have three outlier indicators (i.e., Triple O) and those with two or more “outliers” indicators detected to be true was finally defined as a “brain outlier.”

Based on neonatal functional connectivity data from SAMPLE 1, brain outliers were defined using the proposed Triple O scheme as described above. The correspondence between the detected “brain outliers” and 4YR IQ outliers was examined to test Triple O’s performance in terms of sensitivity, specificity, and overall accuracy. Note for Triple O, all three brain outliers were defined purely based on brain rsfMRI data and no training with 4YR IQ data was needed. Instead, the 4YR IQ outcome data were only used to test the performance of the Triple O model. Therefore, Triple O represents a data‐based prediction scheme with no need of training with outcome data.

### 2.5 Comparison with demographic information‐based outlier detection

Based on the continuous measures of gestational age (GA) at birth, GA at MRI scan/4YR IQ assessment, birthweight, birth length, maternal/paternal age, maternal/paternal education in years, and total annual family income, their correlations with 4YR IQ were calculated. Among all demographic variables, we identified three that (a). we have data on all the 175 subjects in SAMPLE 1; and (b) showed significant positive correlations with 4YR IQ. These three variables were maternal education, birthweight, and gestational age at birth. All three measures were *Z*‐transformed and tested through a similar outlier detection pipeline as did the three brain outliers to define demographic information‐based outliers. The performance of these “demographic outliers” in detecting 4YR IQ outliers was compared with the brain‐based Triple O results.

### 2.6 Demographic information‐enriched Triple O (Triple O+)

To explore whether incorporating demographic information would help specify the polarity of brain‐based outlier prediction, we calculated the average of the three demographics identified as meeting the two criteria above (i.e., GA at birth, birthweight, and maternal education in years) as a Cumulative Demographic Risk Index (CDRI) in SAMPLE 1. Given their consistent positive correlations with 4YR IQ, we predicted that the detected brain “outliers” with above‐average CDRI would correspond to high 4YR IQ performers, while those with below‐average CDRI would correspond to low 4YR IQ performers. Therefore, CDRI was used to further provide a “sign” for Triple O prediction and this enriched model was termed as Triple O+.

### 2.7 Validation based on SAMPLE 2 and its subsamples

To test the robustness of the Triple O+ prediction, the 218‐subject SAMPLE 2 and six subsamples from SAMPLE 2 (i.e., only males (*N* = 108), only females (*N* = 110), data from one of the two scanners (*N* = 181/37 for Scanner 1/2), and two randomly selected 100‐subject subsamples) were used as independent reference samples to identify brain outlier from SAMPLE 1. The corresponding performances were compared with the one using SAMPLE 1 as its own reference to examine Triple O+’s robustness against different reference samples.

### 2.8 The sample size limit and underlying mechanism of Triple O+ prediction

With the validation analyses suggesting that the sample size of the reference group may be a limiting factor for robust performance, we performed a set of random sampling analyses to explore the performance of Triple O+ with different sample sizes of the reference group. Specifically, we randomly selected 10–216 subjects 1,000 times from the SAMPLE 2 (*N* = 218) and tested the prediction performances on the independent SAMPLE 1 (*N* = 175) data. Sensitivity, specificity, and accuracy were calculated at each sample size step. Moreover, we also calculated the mean and standard deviations of the three brain outlier measures (i.e., the number of connection‐level outliers, the Euclidean distance of the overall matrix with the reference group, and the within‐subject standard deviation) to further reveal the underling mechanisms of the performance.

## 3 RESULTS

### 3.1 Participants and 4YR IQ distribution

The detailed demographic information for both SAMPLE 1 and SAMPLE 2 is listed in Table 1. Note both SAMPLE 1 and SAMPLE 2 were composed of mixed samples with respect to sex, term status, twin status, and maternal mental disorder status, and no significant differences were observed against the relative percentages of either of the four categorical variables (Table 1). Moreover, the two samples were also matched against each and every of the 10 continuous demographic variables (i.e., gestational age (GA) at birth, GA at MRI scan/4YR IQ assessment, birthweight, birth length, maternal/paternal age, maternal/paternal education in years, and total annual family income) and no significant difference was observed for any of these variables (Table 1). The mean and standard deviation of the 4YR IQ scores of the 175 study participants in SAMPLE 1 are 106.9 and 11.8, respectively. The actual distribution is shown in Figure 1a (left panel). When compared between females/males, singleton/twin birth, full‐term/preterm, and with/without maternal disorders, significant differences were observed between term–preterm (P/F, *p* = .039) and singleton–twin birth (T/S, *p* = .018), while marginally significant differences were observed between children with and without maternal disorder diagnosis (D/nD, *p* = .103, Figure 1a, middle panel), suggesting that twin status, preterm birth, and maternal disorder diagnosis can be viewed as potential risk factors for lower IQ. Therefore, in the following discussions, we primarily separate SAMPLE 1 into two cohorts: 1. the CONTROL group (i.e., singleton birth, full‐term, and no maternal disorder diagnosis, *N* = 56) and the RISK group (i.e., meeting at least one of the three criteria, including twin, preterm birth, and maternal disorder diagnosis, *N* = 119). When comparing these two groups, their 4YR IQ difference was highly significant (C/R, *p* = 1.379 × 10^–4^; Figure 1a, middle panel). When splitting the whole histogram into the two groups, it is apparent that there were more CONTROLs at the right end (high IQ) but more RISK participants at the left end of the overall 4YR IQ distribution (Figure 1a, right panel). When correlating the 10 continuous demographic variables with 4YR IQ, GA at birth, birthweight, birth length, maternal and paternal education, as well as total annual family income showed significant positive correlations (*r* ~ .17–.40, Figure 1b), supporting reasonable predictive power of these demographic and environmental variables for 4YR IQ.

There were 13 “High IQ outliers” (i.e., IQ > 127, 7.43%, corresponding to the “Superior” and “Gifted” categories in SB5 classification (Roid, 2003)) and 6 “Low IQ outliers” (i.e., IQ < 85, 3.43%, corresponding to the “Low Average” and “Borderline/Mildly impaired or delayed” categories according to SB5 classification (Roid, 2003)). Among the 13 high IQ outliers, 7 were from the CONTROL group and 6 were from the RISK group, while all 6 low IQ outliers were from the RISK group, reaffirming a higher risk of lower 4YR IQ performance from the RISK group. The detailed breakdown of the 13 high/low performers against all demographic categories is listed in Table S1, which shows a mixed distribution, and none of the IQ outliers could be identified simply by his/her demographic classification. Similarly, when the defined IQ outliers were highlighted in the scatter plots of quantitative demographic variable–4YR IQ relationships (Figure 1b), it is apparent that despite significant quantitative correlations with a subset of demographic variables, the IQ outliers could not be readily identified through simple thresholding of any of the demographic variables.

### 3.2 Prediction performance of Triple O

**Figure 2:**
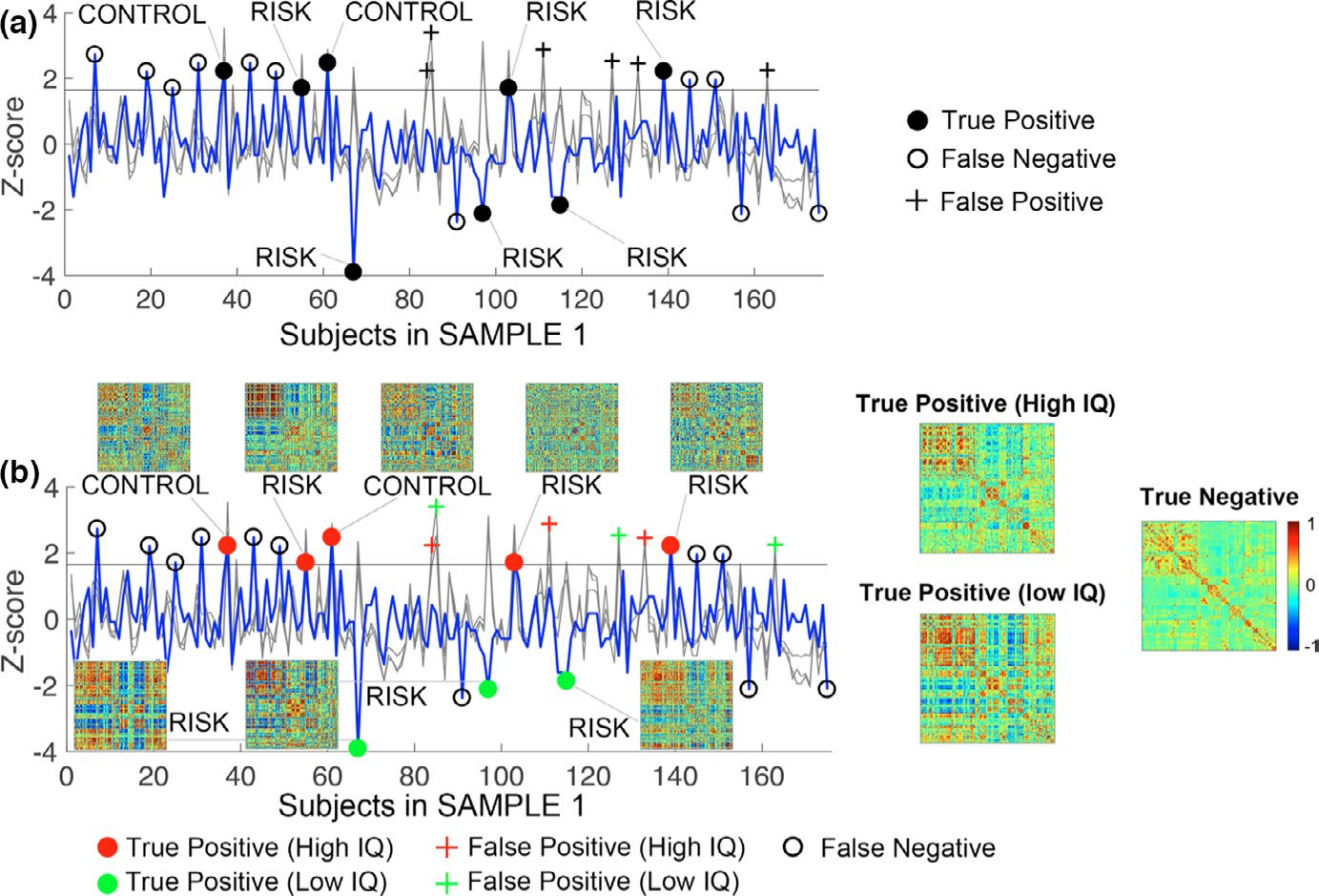
Performances of Triple O and Triple O+ on predicting 4YR IQ outliers based on neonatal functional connectivity outliers. (a) Triple O performance. The *X*‐axis represents individual subjects, while the *Y*‐axis indicates *Z*‐scores of either 4YR IQ performance (blue line) or the three neonatal functional connectivity outlier measures (i.e., Triple O, gray lines). The idea is to examine whether brain outliers (defined as one‐way *t* test of *p* < .05 (the gray horizontal line) for at least 2 out of three brain outlier measures) correspond to 4YR IQ outliers as defined in Figure 1a. As shown in (a), there is a general correspondence between gray line peaks and blue line peaks (either high or low peaks). The solid black dotes indicate true positives (i.e., 4YR IQ outliers that were correctly identified as brain outliers based on Triple O), the crosses indicate false positives (i.e., subjects detected as brain outliers based on Triple O but not 4YR IQ outliers), and the empty circles indicate false negatives (i.e., 4YR IQ outliers that were not detected as brain outliers based on Triple O). The belongings of each true positive to either the CONTROL or RISK group were also noted. (b) Triple O+ performance. Similar curve plots for the three brain outlier measures (gray lines) and their 4YR IQ (blue line) but the detected brain outliers were given either a positive (red) or negative (green) sign depending on whether the individual's Cumulative Demographic Risk Index (CDRI) was above or below the average of the reference group (i.e., incorporating Triple O with CDRI to form Triple O+). Also included in this plot were the individual functional connectivity matrices for the identified true positives, as well as the mean matrices for the true positive (high IQ), true positive (low IQ), and the true negative groups

Triple O’s performance on SAMPLE 1 is shown in Figure 1. The results showed brain outliers defined based on Triple O could identify 8 out of 19 4YR IQ outliers, translating to a 42.1% detection rate (Figure 2a). Specifically, 2 out of 7 high IQ outliers from the CONTROL group (i.e., full‐term, singleton birth, no maternal mental disorder diagnosis), 3 out of 6 high IQ outliers from the RISK group (i.e., 1 preterm, 1 twin birth, 1 preterm + twin birth, Table S1), and 3 out 6 low IQ outliers from the RISK group (i.e., 1 with maternal mental disorder diagnosis, 2 preterm + twin birth, Table S1) were correctly identified based on Triple O (Figure 2a, Table S1). There were also 6 false positives and 11 false negatives, translating to an overall sensitivity of 42.1%, specificity of 96.2%, and accuracy of 90.3% as measured by conventional prediction terms. If we broke down the statistics into CONTROL and RISK groups, the sensitivity/specificity/accuracy for detecting IQ outliers from brain outliers were 28.6%/100%/91.1% for the CONTROL group and 50%/94.4%/89.9% for the RISK group. All three predictions from Triple O (i.e., whole group, within CONTROL and RISK subgroups) were highly significant (*p* < .001) based on Fisher's exact test. However, as expected, Triple O identified both high and low IQ outliers without distinguishing them at this stage.

### 3.3 Prediction performance of Triple O+

The prediction performance of the three demographic variables showing significant positive correlations with 4YR IQ outcomes (i.e., GA at birth, birthweight, and maternal education in years) based on the same Triple O procedure is shown in Figure S1. The results (1 true positive, 8 false positives, and 18 false negatives, Figure S1) are not above chance (*p* = .537) and much worse than the brain‐based “Triple O.”

When we further incorporated the signs of the standardized CDRI scores (i.e., + for above‐average and −for below‐average) into Triple O, the 14 detected brain outliers split into 8 with a positive sign and 6 with a negative sign (Figure 2b). As expected, all 5 true high 4YR IQ outliers were among the positive sign ones and all 3 true low 4YR IQ outliers were among the 6 negative sign ones. Therefore, Triple O+ was able to separate the detected brain outliers into high IQ/low IQ categories with more specific predictions.

In practice, without information from Triple O+ (Table S2a), there would be 12.50%/0% chance for a CONTROL baby to develop high/low outlying 4YR IQ, while there was 5.04%/5.04% chance for a RISK baby to develop high/low outlying 4YR IQ. However, with Triple O+ (Table S2b), for a CONTROL baby, if he/she was detected as a positive brain outlier (i.e., detected as an outlier based on Triple O and with a positive sign on CDRI), then he/she has a 100% chance to develop high 4YR IQ (0% chance to develop low IQ), while if he/she was not detected to be a brain outlier, then his/her chance to develop high 4YR IQ decreases from 12.50% to 9.26%. More importantly, for a RISK baby, if she/he was detected as a positive brain outlier, then she/he has a 50% chance to develop high 4YR IQ (compared to 5.04% without Triple O+) but 0% chance to develop low 4YR IQ based on the Triple O+ prediction (compared to the 5.04% risk without Triple O+). On the other hand, if she/he was detected to be a negative brain outlier (i.e., detected as an outlier based on Triple O and with a negative sign on CDRI), then she/he has a 50% chance to develop low 4YR IQ (compared to 5.04% without Triple O+) and 0% chance to develop high 4YR IQ (compared to 5.04% without Triple O+), representing the case that needs the most attention. Finally, if a RISK baby was not detected as a brain outlier, then his/her chances to develop high/low 4YR IQ were both decreased to 2.80%, compared to 5.04% without Triple O+. Overall, compared to a general 5.04% chance without Triple O+ information, the predicted possibilities for the RISK group to develop either high or low outlying 4YR IQ with Triple O+ improved ~2 (i.e., 5.04% to 2.80%) to ~10 fold (i.e., 5.04%–50%), which may significantly improve clinical decision making in practice.

### 3.4 Robustness of Triple O+ prediction

**Figure 3:**
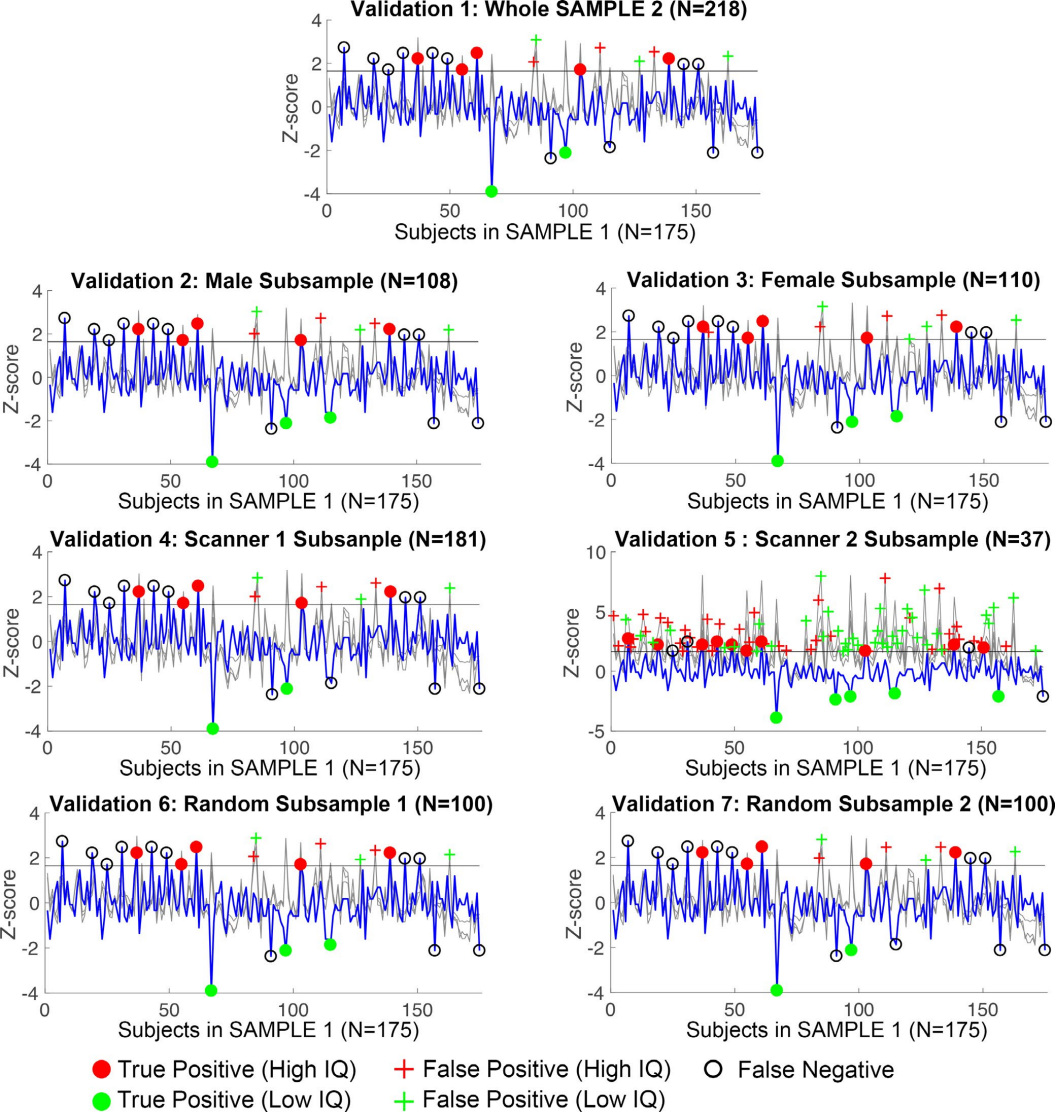
Validation of Triple O+ performances using seven testing reference samples from SAMPLE 2 for prediction of 4YR IQ from SAMPLE 1: including the SAMPLE 2 as a whole, the male/female subsamples, the Scanner 1/2 subsamples, and two random subsamples of 100 subjects. The curve plots are similar to those in Figure 2b

The prediction performances from all seven testing samples (i.e., the whole SAMPLE 2, only males (*N* = 108), only females (*N* = 110), data from one of the two scanners (*N* = 181/37 for Scanner 1/2), and two randomly selected 100‐subject subsamples) are summarized in Table S3. It is striking that 6 out of 7 testing samples produced highly significant (*p* < .001) and consistent prediction performances with those obtained using SAMPLE 1 as its own reference (Figure 3). Specifically, the male‐only subsample (*N* = 108) and the first of the 100‐subject random subsample achieved identical prediction (8/6/11 for true positive/false positive/false negative, with an overall accuracy of 90.3%); the 218 whole sample, the Scanner 1 subsample (*N* = 181), and the second 100‐subject random subsample missed one true positive (7/6/12 for true positive/false positive/false negative, with an overall accuracy of 89.7%), while the female‐only subsample (*N* = 110) produced two more false positives (8/8/11 for true positive/false positive/false negative, with an overall accuracy of 89.1%). The only exception was the Scanner 2 subsample with a much smaller sample size of 37 subjects. When using this small subsample as the reference, Triple O+ caught 15 out of 19 true IQ outliers but also yielded 79 false positives, making it the worst performance (15/79/4 for true positive/false positive/false negative, with an overall accuracy of 52.6%).

### 3.5 The practical limit underlying Triple O+ prediction

**Figure 4:**
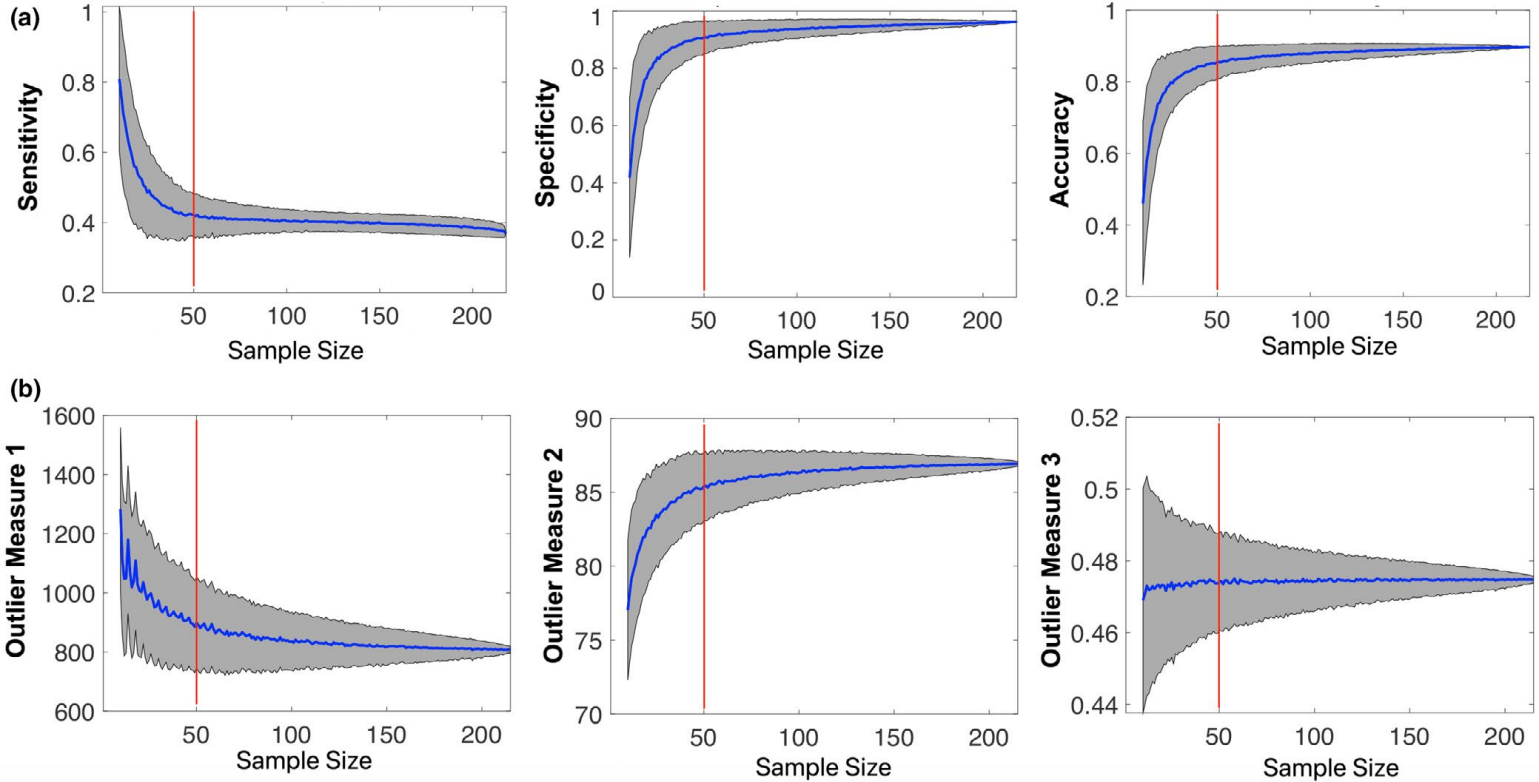
The effects of sample size of the reference group for Triple O prediction performance of 4YR IQ scores. (a) A random resampling (1,000 times at each sample size step) of 10–216 (step size 1) subjects from SAMPLE 2 was done at each step to form the respective reference sample, and the corresponding Triple O performance on predicting 4YR IQ scores in SAMPLE 1 was calculated and shown. (b) The means and standard deviations of the three brain outlier measures for the corresponding reference sample at each step size were calculated and shown. Blue curves represent the mean, while the gray areas represent the standard deviation across the 1,000 random samplings at each sample size step for curves in both (a) and (b). Red line corresponds the step of 50

Results from the testing analyses seemed to suggest that variabilities related to sex and scanner were not significant contributors to prediction performance since the male‐alone, female‐alone, and Scanner 1 subsamples were all able to produce highly consistent prediction results in the mixed testing sample of SAMPLE 1. Indeed, the two randomly selected 100 subsamples also produced equivalent results. However, when the sample size dropped to 37 with the Scanner 2 subsample, performance dramatically degraded. These observations suggested that the sample size of the reference group may be a limiting factor for robust performance. To test this hypothesis and reveal the limit, we randomly selected 10–216 subjects 1,000 times from SAMPLE 2 as the reference sample and tested their prediction performances on SAMPLE 1. As expected, the sensitivity, specificity, and accuracy quickly rose with increasing sample size till around 50, after which all three measures reached plateaus (Figure 4a), suggesting that a sample size of ~50 is needed for a robust prediction based on Triple O. Based on the same resampling, we further calculated the mean and standard deviations of the three brain outlier measures at different sample size steps. It is again apparent that the first two brain outlier measures (i.e., the number of connection‐level outliers and the Euclidean distance of the overall matrix with the reference group) stabilized at around sample size 50 (the third one (i.e., within‐subject standard deviation) showed little changes across the spectrum), providing the underlying functional connectivity stability basis for the observed prediction performance curves.

## 4 DISCUSSION

In this study, we showed that a novel Triple O framework could abstract the neonatal whole‐brain functional connectivity pattern to three *Z*‐scores measuring the degrees of being “outliers” against a reference population. The resulting brain‐based “outlier” indicator could correctly predict 42.1% of 4YR IQ outliers with high specificity (96.2%) and accuracy (90.3%) in 175 subjects with mixed demographic makeup (SAMPLE 1). After combining demographic information, Triple O+ could further differentiate prediction between high and low IQ outliers making it more informative for translational applications. With no need for training, the proposed Triple O+ approach demonstrates high levels of robustness and generalizability underscored by consistent results obtained using a range of independent datasets as the reference sample (i.e., SAMPLE 2 and its five subsamples). Our results further showed a lower limit of 50 on the reference sample size for successful Triple O+ performance.

### 4.1 The importance of early prediction and Triple O/Triple O+ performances

The importance of early identification of risks for adverse developmental outcomes is well agreed upon in the field as the developmental origins of various mental disorders have been increasingly recognized (Monk et al., 2019; Silveira et al., 2007; Swanson & Wadhwa, 2008). The most direct benefits of early prediction include the possibility for early intervention given the widely reported better outcomes associated with earlier initiation of intervention strategies (Guralnick, 2011). In neuroimaging‐based predictions, the neonatal stage may represent the earliest possible timepoint given technical/practical difficulties associated with prenatal MRI imaging (Gao, Lin, Grewen, & Gilmore, 2017), although there are encouraging development in this front in recent years (Thomason et al., 2013). Therefore, the derivation of neonatal brain image‐based prediction schemes, as explored in this study, represents an urgent and important direction that aligns well with our ultimate goal of helping all at‐risk children to reach their greatest potential.

However, one has to recognize that in addition to the neonatal brain developmental status, there are likely a range of other postnatal factors that also contribute to later developmental outcomes (e.g., the 4YR IQ in this study). These may include later structural and functional brain growth, family environment/enrichment activity, parenting, nutrition, adverse life events, among others. Therefore, in the context of this study, one has to be mindful that the neonatal brain may only contain part of the predictive information for 4‐year IQ outcomes, while later brain development and other postnatal environmental factors likely contribute to the rest. As a result, we may not expect that neonatal brain‐based predictions, such as Triple O, to be able to predict all 4YR IQ outliers and this may help explain the 42.1% sensitivity observed in this study. Taking together these considerations, it is actually striking that a simple abstraction scheme such as Triple O, which was based purely on functional connectivity measures at the neonatal stage, could identify >40% of IQ outliers 4 years down the road. This observation reaffirms the developmental cascading hypothesis stating that early brain deviations could cascade and potentially lead to far‐reaching behavioral consequences. We do want to stress that combined with the very high level of specificity (i.e., 96.1%), each and every child identified as a potential “Low IQ outliers” by Triple O likely represents a meaningful risk. If independently validated, Triple O/Triple O+ and their future extensions could significantly aid in clinical decision making, particularly among those infants at higher risk for adverse developmental outcomes (e.g., infants with prenatal drug exposure (Grewen et al., 2020; Salzwedel, Grewen, Goldman, & Gao, 2016; Salzwedel et al., 2015), maternal mental health problems (Qiu et al., 2015), poverty (Kim et al., 2013), and maternal obesity (Salzwedel et al., 2018), among others). Note it is in the RISK group that Triple O actually has a higher detection rate (i.e., 50% in our RISK group compared to 28.6% in our CONTROL group), making its application in at‐risk infant population more justified. Through the earliest possible identification of risks based on Triple O, early intervention becomes possible in this population to potentially rectify early abnormal growth for better outcomes.

Besides postnatal factors beyond the neonatal brain features that may have contributed to 4YR IQ performance, there could be other factors that, if successfully addressed, may help improve the current prediction. These may include (a) other kinds of predictive brain outliers that are not covered by Triple O; and (b) other non‐brain outlier‐based mechanisms/features. Therefore, future studies are needed to explore these possibilities for better prediction. Besides false negatives, the false positives may arise if these subjects possess both “good” outliers and “bad” outliers at the individual connection level whose effects on IQ might cancel each other, thus resulting in “middle” performances. Indeed, when examining the individual and mean group matrices for the high IQ and low IQ outliers, they showed clearly different patterns (Figure 2b). However, the limited sample size from each category prevented us from deriving connectivity signatures for “good” and “bad” outliers. This limitation points to the need for future studies with larger sample sizes to further characterize different types of connection‐level outliers for more accurate prediction.

### 4.2 The rationales behind Triple O/Triple O+

The proposed Triple O approach was inspired by the developmental cascading hypothesis (Masten & Cicchetti, 2010; Monk et al., 2019; Silveira et al., 2007) stating that early changes in the brain could cascade and evolve with age leading to abnormal developmental outcomes. Driven by these hypotheses and our previous empirical results showing significant associations between early functional brain connectivity measures and later behavioral outcomes (Alcauter, Lin, Smith, Short, et al., 2014; Salzwedel et al., 2019), we hypothesize that neonates identified as “brain outliers” would also more likely manifest as “behavioral outliers” four years down the road. This hypothesis serves as the foundation for the Triple O approach. Indeed, the detection of the three low IQ outliers all of whom were in the RISK group, including one with maternal mental disorder diagnosis and two with premature and twin birth status, echoes with this hypothesis and suggests that the genetical factors underlying the disorder diagnosis/potential maternal distress associated with it and premature/twin birth may have contributed to the detected outlying brain connectivity patterns and the subsequent outlying low IQ performance at 4 years of age (Monk et al., 2019). A detailed examination of all eight true positives against the 10 demographic variables failed to show clear signs of extremes that could potentially explain their outlying neonatal brain and 4YR IQ patterns (Figure S2). Future studies with genetic modeling and more comprehensive environmental monitoring are needed to more rigorously examine the potential underlying factors leading to the detected “brain outliers” at the neonatal stage and the later outlying (both high and low) IQ performances.

Empirically, the choices of abstraction measures are almost unlimited, but we chose the three global abstraction measures given the multifaceted nature of IQ performance that covers a multitude of different functional domains (e.g., fluid reasoning, verbal and nonverbal knowledge, quantitative skills, visual–spatial processing, working memory) (Roid, 2003). These functions likely require coordinated functioning of a complex set of distributed functional networks throughout the whole brain (Goriounova & Mansvelder, 2019). Note these three measures were chosen in this proof‐of‐concept study to demonstrate the link between brain outliers and IQ outliers. Future expansions/revisions of the proposed approach, such as including more sophisticated graph theoretical measures (Rubinov & Sporns, 2010), are possible and deserve further exploration. As previously mentioned, another important rationale to use brain outlier measures for prediction relates to its insensitivity to signs of brain–behavioral relationships that are either homogeneous (Alcauter, Lin, Smith, Goldman, et al., 2014; Alcauter, Lin, Smith, Short, et al., 2014; Salzwedel et al., 2019) or heterogeneous (Chen et al., 2020) within the examined population. While prediction schemes directly using functional connectivity strength measures would inevitably be affected/complicated by the different signs of brain–behavior associations between connections and/or the heterogeneity across different subgroups of subjects, the proposed Triple O approach captures the absolute deviations of different functional connectivity strength measures against the population mean regardless of the relative signs and subgroup differences. This sign insensitivity may partly contribute to the observed high level of generalizability to different reference samples as discussed below.

The observed significant quantitative correlations between a range of demographic/participant variables, including parental education, birth outcomes, gestational age, family income, maternal age, and 4YR IQ outcomes (Figure 1b), are consistent with previous findings (Bacharach & Baumeister, 1998; Elgen, Sommerfelt, & Ellertsen, 2003; Eriksen et al., 2013; Tong, Baghurst, Vimpani, & McMichael, 2007). Leveraging these quantitative associations, we derived a Cumulative Demographic Risk Index (CDRI) and predicted that the detected brain “outliers” with above‐average CDRI would correspond to high 4YR IQ outliers, while those with below‐average CDRI would correspond to low 4YR IQ outliers. Our results confirmed this prediction, and the addition of the CDRI indicator enabled the resulting Triple O+ approach to distinguish between high and low IQ outliers (Figure 2b). Therefore, by combining Triple O with demographic information as implemented in Triple O+, the translational potential of the proposed abstraction scheme is further improved by the identification of those at risk to develop as low IQ outliers. As mentioned above, the identification of risks for low IQ outliers may be especially important and productive among vulnerable infant populations including those with prenatal drug exposure, maternal mental health problems, poverty, maternal obesity, among others. For the CONTROL group, since none of them develop as low IQ outliers in SAMPLE 1, the main benefit is the knowledge that the detected “brain outliers” have a higher chance of developing as high IQ outliers (Figure 2b). This information, although not as clinically critical as predictions of low IQ outliers, could still be helpful and beneficial to identify potential gifted children.

### 4.3 The high level of robustness and generalizability of Triple O+

The Triple O+ approach proposed in this study comes from a hypothesis‐driven understanding of the infant brain and brain–behavioral relationships specifically designed to promote robustness and generalizability of prediction. In particular, the three most salient features of Triple O lie in its high level of abstraction, insensitivity to signs of brain–behavioral associations either homogenous or heterogeneous within the examined population, and no need for training with behavioral outcome data. All three features promote robustness and generalizability and could potentially avoid the commonly encountered “overfitting” problem in training‐based machine learning approaches. Indeed, highly consistent prediction performances were observed when using the independent SAMPLE 2 data as the reference sample. Importantly, even when using the male‐only or female‐only subsamples within SAMPLE 2 as reference, highly consistent predictions were observed for SAMPLE 1 with mixed sexes, suggesting that the proposed scheme is not sex‐dependent. Moreover, similar prediction performances could be achieved even when using data from one scanner to predict data from two scanners. These observations are encouraging and support future cross‐institutional applications of Triple O. To add to its practical applications, a low limit of 50 in sample size was demonstrated to achieve similar performances as shown in Figure 4. Taken together, the demonstrated robustness and a relatively minimal requirement of sample size (i.e., ~50) support Triple O as a promising and practical way of identifying newborns at risk for adverse IQ outcomes.

### 4.4 Limitations

Several additional limitations of this study are worth discussing. The first one reiterates the undetected 4YR IQ outliers. Other types of brain outliers, non‐outlier‐based brain mechanisms, and postnatal factors (e.g., brain development, family environment, adverse life events, education, and nutrition) could all underlie this observation, and future studies are needed to explore/validate these possibilities. In particular, future studies further incorporating postnatal environmental factors may prove particularly effective for better prediction but this inclusion may inevitably delay the timing of prediction and considerations have to be given to balance between higher sensitivity and earlier detection. Related, in the current study, the demographic information was reduced to a sign indicator and incorporated in our Triple O+ to only help identify the sign of IQ outliers (i.e., high and low IQ outliers) but future efforts are needed to further explore best ways to incorporate quantitative demographic information for potential improvement in prediction sensitivity/accuracy. The second limitation relates to the relatively small number of IQ outliers (19 from 175) for prediction, reflecting a limited degree of variability in 4YR IQ outcomes in the current population. Future studies with a larger sample size and/or a larger degree of IQ variability are needed to independently validate the current results. Moreover, our validation SAMPLE 2 does not have 4YR IQ data, which prevented us from performing a complete validation test within SAMPLE 2. Future studies with samples that have both neonatal brain and 4YR IQ data are thus needed to further validate the current findings. In addition, efforts could be spent on distinguishing between “good” and “bad” functional connectivity outliers for potential improvement in prediction. Third, we only tested the performance of Triple O/Triple O+ on 4YR IQ outcome measures. Future efforts are needed to test whether variants of Triple O/Triple O+ could predict other domains of behavior outcomes at the same or other ages. Finally, although our tests on male‐only, female‐only, and one scanner‐only reference samples revealed highly consistent results (as long as sample sizes are larger than 50) promoting cross‐institutional application of Triple O+, actual tests with data from different institutions are needed to confirm this potential.

## 5 CONCLUSIONS

In conclusion, we propose a Triple O+ approach to use brain‐based outlier indicators in neonates, enriched by demographic information, to predict high and low outlying IQ performers at 4 years of age. Our results revealed an 42.1% identification rate of 4YR IQ outliers (i.e., 8 out of 19) among a mixed cohort of 175 newborns with differential term, twin, and maternal disorder statuses. Together with a high specificity of 96.2%, Triple O reached an overall accuracy of 90.3% in identifying 4YR IQ outliers. High levels of robustness and generalizability were also observed when using independent datasets as the reference samples. Featured by no need for training, a “small‐data” requirement (lower limit of 50), straightforward interpretations, and high levels of robustness and generalizability, Triple O+ may have the potential for translational applications as a novel way for brain‐based identification of newborns at risk for adverse IQ outcomes years down the road. However, one should also be mindful of the ~60% 4YR IQ outliers that were not detected using Triple O based on the neonatal functional connectivity data. Other types of neonatal brain outliers, other‐than‐outlier‐based mechanisms, postnatal brain growth, or other postnatal family/environmental factors could all have contributed to these false negatives, and future efforts are needed to improve upon Triple O for a higher rate of risk identification.

## CONFLICT OF INTEREST

The authors declare no conflict of interest.

## AUTHOR CONTRIBUTION

WG conceived the Triple O idea. WG and JHG designed study and performed research. YC and EC analyzed data. WG wrote the paper with input from YC, EC, BDG, and JHG.

## Supporting information

Supinfo

## Data Availability

The data presented in this paper and the code to generate these results will be made available upon reasonable request.
