# Supplementary material for: Neonatal brain connectivity outliers identify over forty percent of IQ outliers at 4 years of age
Source: Brain Behav. 2020 Sep 17;10(12):e01846. doi: 10.1002/brb3.1846 (PMC7749582; doi:10.1002/brb3.1846)
Supplement: Supplementary file 1 — Supinfo [file BRB3-10-e01846-s001.docx]

**Supporting Materials:**

**Supporting Tables:**

**Table S1. Distribution of identified 4YR IQ “outliers” in different demographic categories.**

|  | **CONTROL** | **Preterm**  **(P)** | **Maternal**  **Disorder**  **(D)** | **Twin**  **(T)** | **P+D** | **P+T** | **D+T** | **Total** |
| --- | --- | --- | --- | --- | --- | --- | --- | --- |
| **High IQ Outliers** | 7(2) | 1(1) | 1(0) | 3(1) | 0 (0) | 1(1) | NA | 13(5) |
| **Low IQ Outliers** | 0 (0) | 0 (0) | 1(1) | 2(0) | 0 (0) | 3(2) | NA | 6(3) |
| **Total N in each group** | 56 | 4 | 18 | 38 | 3 | 56 | NA | 175 |
| **High IQ Outlier**  **Percentage** | 12.50% | 25.00% | 5.56% | 10.71% | 0% | 1.79% | NA |  |
| **Low IQ Outlier**  **Percentage** | 0% | 0% | 5.56% | 2.63% | 0% | 5.36% | NA |  |

**Numbers in parenthesis indicate the ones that were identified as neonatal brain “outliers” based on Triple O.**

**Table S2a. 4YR IQ outlier probability without “Triple O+” information**

|  | **% Low IQ** | **% High IQ** |
| --- | --- | --- |
| **CONTROL** | 0% | 7/56=**12.50%** |
| **RISK** | 6/119=**5.04%** | 6/119=**5.04%** |

**Table S2b. 4YR IQ outlier probability with “Triple O+” information**

|  |  | **% Low IQ Outlier** | **% High IQ Outlier** |
| --- | --- | --- | --- |
| **CONTROL** | **+Brain outliers** | 0% | 2/2=**100%** |
|  | **-Brain outliers** | Non-detected | Non-detected |
|  | **Non-Brain outliers** | 0% | 5/54=**9.26%** |
| **RISK** | **+Brain outliers** | 0% | 3/6=**50%** |
|  | **-Brain outliers** | 3/6=**50%** | 0% |
|  | **Non-Brain outliers** | 3/107= **2.80%** | 3/107=**2.80%** |

**Table S3. The prediction performances for 7 validation tests**

|  | **SAMPLE2**  **N=218** | **Male**  **N=108** | **Female**  **N=110** | **Scanner1**  **N=181** | **Scanner2**  **N=37** | **Random**  **Subsample1**  **N=100** | **Random**  **Subsample2**  **N=100** |
| --- | --- | --- | --- | --- | --- | --- | --- |
| **TP/FP/FN*** | 7/6/12 | 8/6/11 | 8/8/11 | 7/6/12 | 15/79/4 | 8/6/11 | 7/6/12 |
| **Sensitivity** | 0.368 | 0.421 | 0.421 | 0.368 | 0.790 | 0.421 | 0.368 |
| **Specificity** | 0.962 | 0.962 | 0.949 | 0.962 | 0.494 | 0.962 | 0.962 |
| **accuracy** | 0.897 | 0.903 | 0.891 | 0.897 | 0.526 | 0.903 | 0.897 |
| **p-value** | 0.000 | 0.000 | 0.000 | 0.000 | 0.027 | 0.000 | 0.000 |

*TP: true positive; FP: false positive; FN: false negative.

**Supporting Figures:**

demographic variable. The solid ones represent the ones detected by Triple O (i.e., true positives) while the empty circles represent those not.

**Supporting Figures:**


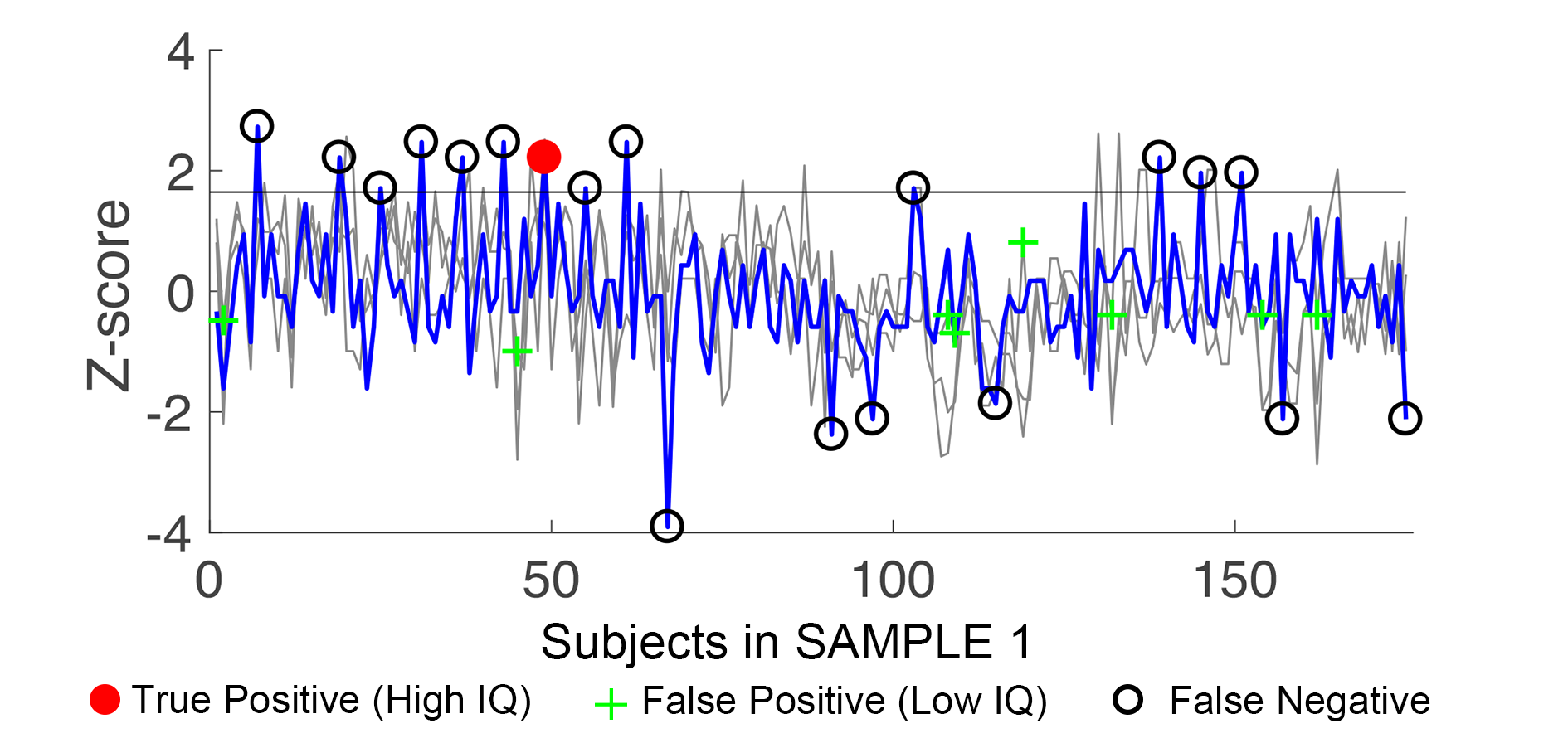


**Figure S1. The prediction performance based on the demography information using similar Triple O pipeline. There were 1 true positive, 8 false positives, and 18 false negatives and the performance is not significant (p=0.537).**


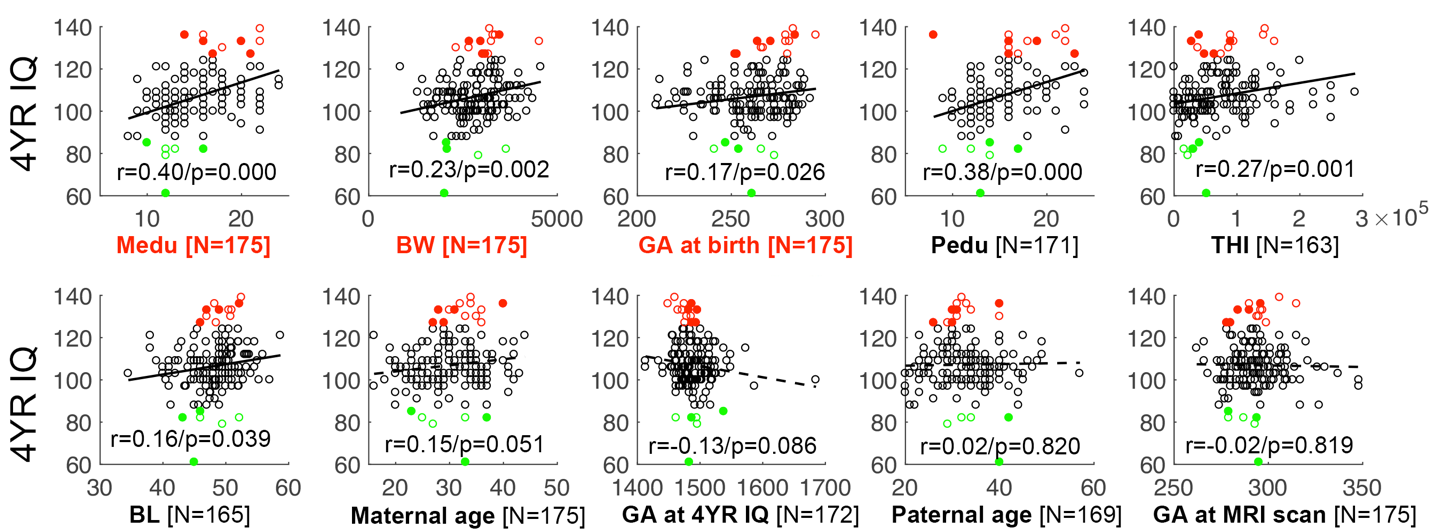


**Figure S2. Re-plots of the scatter plots in Fig. 2b (i.e., correlations between all continuous demographic variables and 4YR IQ) but highlighting the detected true positives (solid red/green dots for high/low IQ outliers, respectively; other 4YR IQ outliers not detected by Triple O were shown in empty red/green dots) against each demographic variable to examine if they show extreme values in any of these demographic domains. As shown, there is no clear trends of extreme demographic values associated with the detected true positives through Triple O.**
